# Long Term 5-Year Survival of Persons with Cryptococcal Meningitis or Asymptomatic Subclinical Antigenemia in Uganda

**DOI:** 10.1371/journal.pone.0051291

**Published:** 2012-12-10

**Authors:** Elissa K. Butler, David R. Boulware, Paul R. Bohjanen, David B. Meya

**Affiliations:** 1 Department of Medicine, University of Minnesota, Minneapolis, Minnesota, United States of America; 2 Infectious Disease Institute, Makerere University, Kampala, Uganda; 3 Department of Medicine, Makerere University College of Health Sciences, Kampala, Uganda; University of Ottawa, Canada

## Abstract

Data presented previously as an abstract at the 2011 CUGH Global Health Conference in Montreal, Canada on 15 Nov 2011. The long-term survival of HIV-infected persons with symptomatic cryptococcal meningitis and asymptomatic, subclinical cryptococcal antigenemia (CRAG+) is unknown. We prospectively enrolled 25 asymptomatic, antiretroviral therapy (ART)-naïve CRAG+ Ugandans with CD4<100 cells/mcL who received pre-emptive fluconazole treatment (CRAG+ cohort) and 189 ART-naïve Ugandans with symptomatic cryptococcal meningitis treated with amphotericin (CM cohort). The 10-week survival was 84% (95%CI: 70–98%) in the CRAG+ cohort and 57% (95%CI: 50%–64%) in the CM cohort. The CRAG+ cohort had improved five-year survival of 76% (95%CI: 59%–93%) compared to 42% (95%CI: 35%–50%) in the CM cohort (*P* = 0.001). The two cohorts had similar immunosuppression pre-ART with median CD4 counts of 15 vs. 21 CD4/mcL in the CRAG+ and CM cohorts, respectively (*P* = 0.45). Despite substantial early mortality, subsequent 5-year survival of persons surviving 6-months was excellent (>88%), demonstrating that long term survival is possible in resource-limited settings. Pre-ART CRAG screening with preemptive fluconazole treatment and improved CM treatment(s) are needed to reduce AIDS-attributable mortality due to cryptococcosis which remains 20–25% in sub-Saharan Africa.

## Introduction

Cryptococcal meningitis (CM) is responsible for approximately 20–25% of AIDS-attributable mortality in sub-Saharan Africa [1], [2]. Although early antiretroviral therapy (ART) prevents cryptococcosis, only three countries in Africa have accomplished >80% ART coverage. Thus, 1.8 million HIV-infected persons still died worldwide in 2010 [3]. The annual incidence of CM in sub-Saharan Africa is estimated at ∼720,000 cases with hundreds of thousands of deaths annually [1]. The cryptococcal antigen (CRAG) is a highly sensitive and specific test in serum or plasma for detecting cryptococcal infection in AIDS patients, even in asymptomatic persons with subclinical infection. Pre-ART serum CRAG positivity is 100% sensitive and CRAG titers above 1∶8 are 96% specific for predicting later development of CM during the first year of ART in asymptomatic persons with CD4 counts ≤100 cells/µL [4]. In persons with AIDS, CRAG is positive in serum at least a median of 22 days before symptoms and signs of cryptococcal meningitis become apparent [4]–[6]. The prevalence of cryptococcal antigenemia in HIV-infected persons with CD4 counts <100 cells/µL ranges from 2.5% to 12% in Sub-Saharan Africa and Southeast Asia [6], and cryptococcal antigen (CRAG) screening followed by preemptive treatment is cost saving in high-risk populations [6]–[8].

Among persons presenting with symptomatic CM, even with optimal treatment with amphotericin B and antiretroviral therapy (ART), mortality from CM remains high (30–50%) [9]–[11]. The long-term survival of persons with symptomatic cryptococcosis on ART has not been evaluated. The majority of studies assessing outcomes of cryptococcal meningitis have focused on 10-week survival [12]–[15]. In this study, comparing two previously described cohorts [6], [16], [17], we report the ongoing long term 5-year survival of Ugandans with AIDS and either asymptomatic cryptococcal antigenemia or symptomatic CM.

## Methods

HIV-infected subjects were enrolled in two prospective cohorts at the Infectious Disease Institute in Kampala, Uganda. The first cohort, as previously described [6], included 25 persons with asymptomatic cryptococcal antigenemia (CRAG+ cohort) who were identified through screening of 609 persons with CD4<200 cells/µL initiating ART in the clinic during 2004–2007 and who received preemptive fluconazole treatment (asymptomatic CRAG+ cohort) [6]. Of the 609 persons screened, 50 (8.2%) were CRAG positive. CRAG+ persons with a remote history of treated CM (n = 17) were excluded from this analysis. Among persons with CD4≤100, the prevalence of incident CRAG+ was 8.8%. Eight CRAG+ asymptomatic subjects not receiving fluconazole preemptive treatment (25% survival at 2.5 year follow up) were excluded. The second cohort consisted of 189 ART-naïve subjects with symptomatic CM recruited from the inpatient infectious disease ward at Mulago Hospital and enrolled from 2006–2009, as previously described (CM cohort) [17]. Inclusion criteria for both studies were (1) confirmed HIV-1, (2) stable residence within ≤20 km, (3) willingness to exclusively receive HIV care at the clinic for ≥2 years, (4) eligibility for ART according to the World Health Organization (WHO) 2003 guidelines and the Uganda National Ministry of Health Guidelines, and (5) provision of written informed consent. The CM cohort had diagnoses by CSF culture and/or CSF CRAG latex agglutination. CD4 and viral load testing were performed pre-ART in the outpatient clinic. The ethics committees of Makerere University and Uganda National Council for Science and Technology approved this research.

Those in the asymptomatic CRAG+ cohort were treated with fluconazole with doses ranging from 200–400 mg daily (400 mg n = 22, 200 mg n = 6, unknown dose n = 3 but ≤400 mg) for 2–4 weeks (4-weeks n = 7, 2-weeks n = 8, 1-week n = 1, unknown duration n = 9 but ≤4 weeks). At the time, no guidelines existed for preemptive treatment of asymptomatic cryptococcal antigenemia; thus, fluconazole dosage and treatment duration were at physician discretion. Subjects began ART within ∼1 week of CRAG screening. Subjects in the symptomatic CM cohort were treated with 50 mg IV amphotericin B (approx. 0.7–1 mg/kg/day) for 14 days followed by oral fluconazole at 400 mg/day for 8 weeks, and then received secondary prophylaxis with 200 mg/day of fluconazole indefinitely. These subjects began ART at the first outpatient visit 4–5 weeks after discharge from the hospital. Among 95% of subjects with CM, this was their first AIDS-defining illness and new HIV diagnosis. Subjects were followed at regular intervals until end of life, transfer of care, or loss to follow up.

We assessed survival via Kaplan-Meier curve with comparison via Log Rank test of the two cohorts. Explanatory variables were evaluated using Cox proportional hazards regression analysis (SPSS 19.0.2, IBM). For demographics, Fischer’s exact Chi-Square test compared categorical variables. Means were compared by Student *t*-test, and medians were compared by the Mann-Whitney U test.

## Results

### Cohort Characteristics

Twenty-five asymptomatic CRAG+ persons were enrolled into the CRAG+ cohort and 189 persons with symptomatic CM were enrolled into the CM cohort. Of the 214 persons enrolled in the two cohorts, demographic and clinical data are presented in Table 1. The mean age of persons was 36±9 years and 118 (55%) were male. Subjects had a mean BMI of 19±3 kg/m^2^. The median CD4 cell count was 20 cells/µL (interquartile range (IQR): 7–45 cells/µL) and median viral load was 5.3 log_10_ copies/mL (IQR: 5.0–5.8 log_10_ copies/mL). Fifteen (60%) persons in the asymptomatic CRAG+ cohort were categorized with WHO clinical stage 3 disease, and all persons with CM, by definition, had WHO clinical stage 4 disease. At pre-ART, the CRAG+ cohort had slightly higher viral loads (*P* = 0.001), and similar CD4 counts (*P* = 0.45) compared to the CM cohort.

**Table 1:** Characteristics of persons presenting with asymptomatic, subclinical cryptococcal antigenemia (CRAG+) and symptomatic cryptococcal meningitis.

Variable	Asymptomatic CRAG+(n = 25)	Cryptococcal Meningitis(n = 189)^a^	*P*
Mean Age – years (±SD)	35.7 (±7.2)	36.2 (±8.8)	.75
Male Sex – no. (%)	11 (44%)	107 (56%)	.29
Mean BMI – kg/m^2^ (±SD)	19.4 (±3.1)	19.1 (±2.9)	.63
WHO Clinical Stage of HIV/AIDS – no. (%)			<.001
WHO Clinical Stage 3	15 (60%)	0 (0%)	
WHO Clinical Stage 4	10 (40%)	189(100%)	
Median Pre-ART CD4 count – cells/µL (IQR)	15 (4–57)	21 (8–42)	.45
Median CD4 count at 6 months – cells/µL (IQR)	146 (110–241)	103 (67–159)	.001
Median Pre-ART Viral Load – log_10_ copies/mL (IQR)	5.8 (5.3–5.9)	5.3 (4.8–5.7)	<.001

a Pre-ART CD4 and viral load data were only available for 117 symptomatic CM persons, collected pre-ART.

### Survival

**Figure 1:**
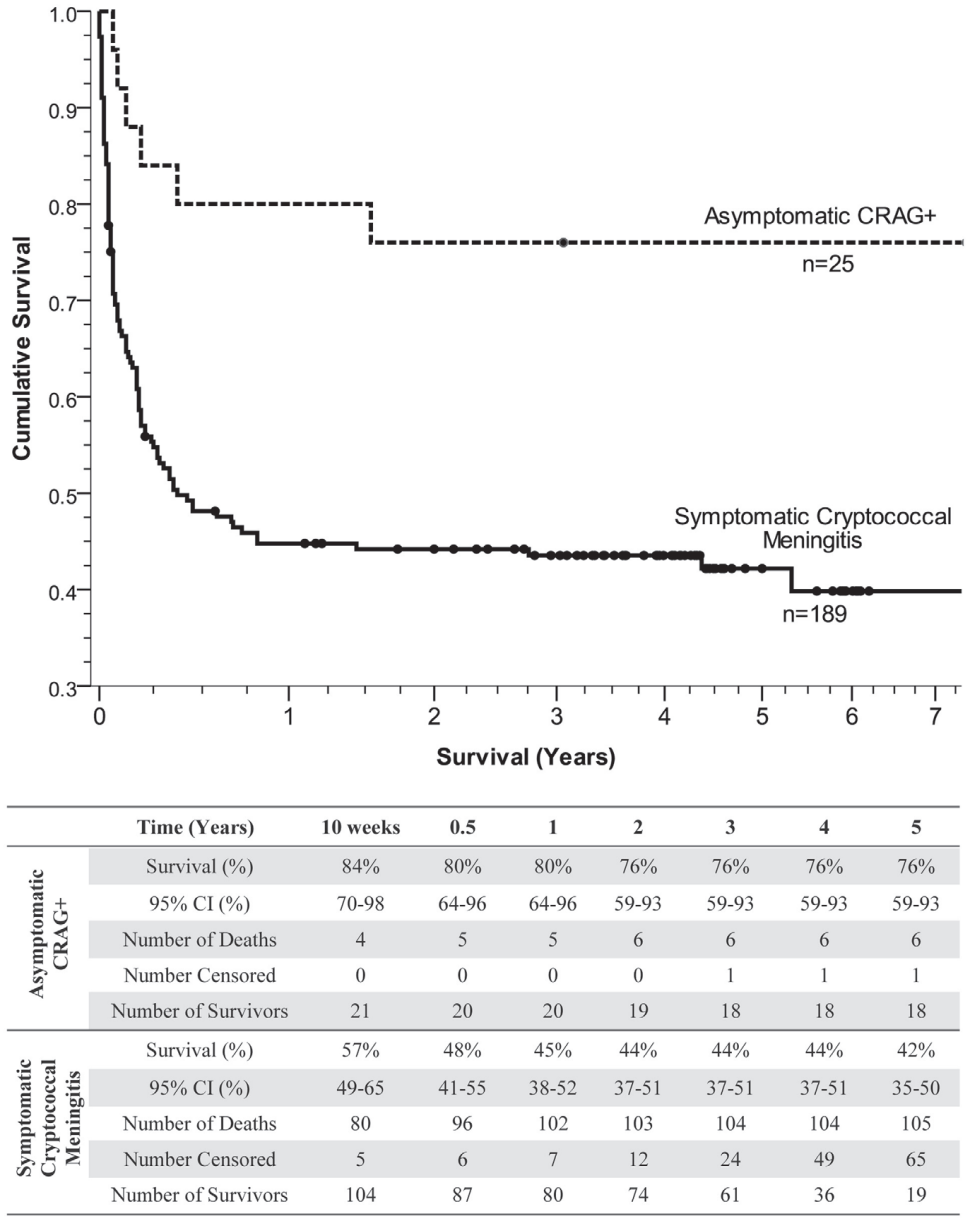
Long term survival among HIV-infected persons with cryptococcosis in Uganda. The Kaplan-Meier survival curve displays the long term survival of 25 asymptomatic persons who tested positive for serum cryptococcal antigen (CRAG+) treated with fluconazole and ART compared to 189 symptomatic patients with cryptococcal meningitis (CM) treated with amphotericin B induction and then fluconazole consolidation therapy and then ART. Diamonds represent censored data. Persons were right-hand censored if they were still living at the time of analysis (n = 82), if they transferred their care to another clinic (n = 7), or if they were lost to follow up (n = 9). Survival table showing survival rate, number of deaths, number censored, and number of survivors at yearly intervals for each cohort.

The Kaplan-Meier method estimated survival for each cohort (Figure 1). The start time for analysis for persons in the asymptomatic CRAG+ cohort was the date of CRAG screening, which was ∼1 week prior to ART initiation. For the CM cohort, the start time was the date of CM diagnosis, and ART was initiated a median of 5 weeks after CM diagnosis, which is consistent with WHO guidelines. Persons were right-hand censored if they were still living at the time of analysis (n = 82), if they transferred their care to another clinic (n = 7), or if they were lost to follow up (n = 9). All requirements for survival analysis were met. The major difference in mortality between the two cohorts was due to the acute mortality associated with CM, particularly in the first four weeks. In the CRAG+ cohort, the 10-week survival was 84% (95% CI: 70% to 98%), and in the CM cohort, the 10-week survival was 57% (95% CI: 50% to 64%). The one-year survival was 80% (95% CI: 64% to 96%) for the asymptomatic CRAG+ cohort and 45% (95% CI: 37% to 52%) for the CM cohort. At 5 years, the asymptomatic CRAG+ cohort had a survival of 76% (95% CI: 59% to 93%), and the CM cohort had a survival of 42% (95% CI: 35% to 49%) (Log Rank test: *P*  = 0.001). Of those with CM surviving to start ART, 6-month survival on ART was 65% (95% CI: 55% to 75%). Among those who survived 6 months on ART, the 5-year survival thereafter was 95% (95% CI: 86% to 100%) for the asymptomatic CRAG+ cohort and 88% (95% CI: 79% to 96%) for the CM cohort (*P*  = 0.23).

### Evaluation of Explanatory Variables

Evaluation of cohort, sex, age, BMI, baseline CD4 count, and viral load as explanatory variables in the Cox proportional hazards regression analysis revealed that only the presence of symptomatic CM was a significant predictor of mortality (hazard ratio  = 3.58, 95% CI: 1.55 to 8.27). In follow up at 6-months, the asymptomatic CRAG+ cohort had a higher median CD4 count as compared to the CM cohort (146 vs. 103 cells/µL, *P*  = 0.001).

## Discussion

We describe the 5-year survival of those with asymptomatic, subclinical CRAG antigenemia and those with symptomatic CM. This is the first study to evaluate long-term survival at 5 years in persons with subclinical or overt cryptococcosis in sub-Saharan Africa. Previous studies assessing outcomes of CM showed poor short-term survival. Most of these studies investigated 2 and 10-week survival [12]–[15], [18], [19], and only four cohorts have evaluated survival at 6 months [20] or ≥1 year [16], [21], [22]. Like previous studies, our results showed high early mortality, with highest mortality in the first 6 months for both cohorts. Even after ART initiation, the mortality after CM in Uganda was higher than previously reported in Thailand during the first 6-months of ART (35% vs. 5%, respectively), although the timing of ART initiation differed (median: 5 weeks in Uganda vs. 11 weeks in Thailand) [21], [23]. The long-term 5-year survival among those surviving 6-months was excellent (>88%) in Uganda, even for persons with symptomatic CM. These results are similar to the prior Thai experience [21], but our study shows that the survival benefit of ART extends five years or beyond. Thus, our long-term study provides valuable outcome data that can be utilized for cost-effectiveness analysis research to understand how enhancement of initial cryptococcal care to improve short term outcomes likely translates to improving long-term outcomes. The survival after 6-months that we observed is similar to a large Uganda cohort with AIDS without cryptococcosis [24]. Additional research is needed to improve the short-term survival of patients with CM, and ultimately translate this into improved long-term survival.

A better overall strategy to improving outcomes, however, is avoiding the development of symptomatic CM. Our data indicate that preemptive treatment of asymptomatic cryptococcal antigenemia was associated with improved survival as compared to treatment of persons with symptomatic meningitis, despite similar levels of advanced immunosuppression. We identified the presence of symptomatic CM as the only predictor of mortality when sex, age, BMI, baseline CD4 count, and viral load were considered.

Jarvis *et al*, Meya *et al*, and Pongsai *et al* have previously demonstrated the need for CRAG screening in persons with CD4 counts of ≤100 cells/µL [4], [6], [25]._ENREF_16_ENREF_17 Meya *et al* calculated that 11.3 persons with advanced immunosuppression need to be screened to detect 1 case of cryptococcal antigenemia in Kampala, Uganda [6]. Based on the Jarvis *et al* calculation of positive predictive value of CRAG+ for progression to CM (29%) [4], 66.7 persons with CD4≤100 cells/µL would need to be screened and, those found to be positive, treated to prevent one case of CM. Differences in disease progression between the CRAG+ cohorts may relate to differences in the median CD4 T cell counts of 15 cells/µL in Uganda vs. 46 cells/µL in South Africa and/or differences in CRAG titers between cohorts (unknown) [4], [6], [8]. What is the exact cost-effectiveness of CRAG screening and preemptive therapy, likely varies by locale. Our long-term survival data presented here further reinforce the need for implementation of CRAG screening in persons with advanced HIV infection in sub-Saharan Africa. Serum/plasma CRAG+ individuals can then be effectively preemptively treated before development of symptomatic CM [4], improving long-term outcomes in a likely cost-effective manner [8], [26], [27]. Clinical algorithms for implementation of cryptococcal screening have been published [26], [28].

Because in our CRAG+ cohort the dosage and duration of preemptive anti-fungal therapy was variable and left to the discretion of the treating physician, the optimal preemptive treatment regimen for CRAG+ persons remains unknown. The December 2011 WHO rapid advice on cryptococcal disease conditionally recommends screening for all ART-naïve patients with CD4≤100 cells/µL where the prevalence of cryptococcal antigenemia is ≥3% [29]. The WHO recommended treatment of asymptomatic CRAG+ persons with fluconazole 800 mg/day for 2-weeks, followed by 400 mg/day for 8-weeks, and then maintenance therapy of 200 mg/day for at least 1 year coupled with ART initiation after 4 weeks of antifungal therapy [29]. This conditional recommendation is based on expert opinion and prospective studies are urgently needed to determine optimum treatment. Our data suggest that late cryptococcal relapse is uncommon in subclinical CRAG+ receiving preemptive fluconazole and that long term secondary fluconazole prophylaxis may be unnecessary for most. More data are urgently needed.

This study is limited because complete demographic data, such as CD4 count and viral load were collected pre-ART, and thereby were missing from 72 patients with CM who died in the hospital before time of clinic registration. This lack of information did not influence our survival analysis, and based on the distribution of CD4 data for persons with and without CM (median CD4 of 21 and 15 cells/µL, respectively), was unlikely to fundamentally alter our conclusions. Baseline CD4 count has not been associated with in-hospital or pre-ART survival after CM [21], [30], [31], likely because all of these patients have very low CD4 counts. In our study, if one imputed missing CD4 values of all those with CM who died pre-ART as 5 cells/µL, there still would not be a statistical difference in CD4 counts between the CRAG+ and CM cohorts (*P* = 0.52). Both groups had very advanced immunosuppression and would have been at risk of other AIDS-related causes of mortality before presenting to medical care. Thus, these data cannot inform the mortality prior to presenting to healthcare. The 5-year lost to follow up rate we observed was only 4.2% (9/214); thus, we believe the survival data are robust, correct, and likely generalizable of long term ART outcomes in well run ART clinics. Data on concurrent other opportunistic infections, which could possibly be an additional explanatory variable, were unavailable for all subjects; however, because both cohorts had similar levels of immunosuppression, it is unlikely that there was a difference in distribution of co-infections between the two cohorts. Additionally ART start time and slight differences in enrollment periods for each cohort could be explanatory in the survival benefit. The difference in ART start time does not significantly affect our results because early ART initiation after CM is likely harmful (unpublished, NCT01075152). The difference in enrollment periods if anything, underestimates the survival benefit of screening and preemptive treatment.

One fundamental conclusion is medical treatment of CRAG antigenemia, a subclinical pre-disease state, results in better outcomes than treatment of overt clinical disease due to CM. While this is an obvious conclusion, a second, perhaps more important, conclusion is that long term survival among those surviving 6-months is excellent (>88%), suggesting that improvement in short term survival through CRAG screening or better CM treatment will translate into long term outcome benefits. Ultimately, cryptococcosis will be drastically reduced by earlier HIV testing, retention-in-care, and universal access to ART. However, in areas that continue to have suboptimal ART coverage, two further interventions should be pursued by stakeholders. First, preemptive treatment of asymptomatic CRAG+ persons with advanced AIDS identified by CRAG screening will result in improved long-term survival and prevent the high human and economic costs of overt cryptococcal meningitis. Second, more resources allocated toward better CM treatment could promote improved short term survival which would translate into long-term survival. This manuscript demonstrates that long term survival after cryptococcosis is possible with ART in resource-limited settings.
